# Radiographic and MR Imaging Findings of the Spine after Bisphosphonate Treatment, in a Child with Idiopathic Juvenile Osteoporosis

**DOI:** 10.1155/2015/727510

**Published:** 2015-01-20

**Authors:** Olympia Papakonstantinou, Maria Sakalidou, Erato Atsali, Vasiliki Bizimi, Maria Mendrinou, Efthymia Alexopoulou

**Affiliations:** ^1^2nd Department of Radiology, “Attikon” University Hospital, National and Kapodistrian University of Athens, Rimini 1, Chaidari, 124 62 Athens, Greece; ^2^3rd Department of Pediatrics, “Attikon” University Hospital, Chaidari, 124 62 Athens, Greece

## Abstract

Bisphosphonates are employed with increasing frequency in various pediatric disorders, mainly associated with osteoporosis. After cessation of bisphosphonate treatment in children, skeletal radiologic changes have been documented including dense metaphyseal lines of the long bones and “bone in bone” appearance of the vertebrae. However, the evolution of these radiographic changes has not been fully explored. We describe the MR imaging appearance of the spine that, to our knowledge, has not been previously addressed in a child with idiopathic juvenile osteoporosis who had received bisphosphonates and emphasize the evolution of the radiographic findings of the spine and pelvis over a four-year period.

## 1. Introduction

Idiopathic juvenile osteoporosis (IJO) is a self-limited disorder that occurs sporadically in children with no family history of bone disease and is a diagnosis of exclusion [1]. Children characteristically refer to bone pain and fractures of long bones and vertebrae following minor trauma whereas bone mineral content (BMC) and density (BMD) are low. Recurrent fractures may lead to kyphosis and severe skeletal deformities [2, 3].

During the last years treatment with bisphosphonates has emerged in the therapeutic management of children with primary osteoporosis mainly suffering from osteogenesis imperfecta or secondary osteoporosis of various etiologies [1–4]. Despite limited efficacy data on the employment of bisphosphonates in IJO, there is firm evidence that bisphosphonate administration in patients with symptomatic IJO leads to marked clinical improvement [1–4]. Previous studies have documented the development of dense metaphyseal lines after bisphosphonate administration in children and a “bone in bone” or a “picture frame” appearance of vertebral bodies [4–11], although the association between the evolution of radiographic changes in the spine and those in long and flat bones has not been adequately explored. In addition, to our knowledge, the MR imaging findings of the spine after bisphosphonate treatment in children have not been presented.

We aim to describe the evolution of the radiographic changes in the spine and pelvis, over a four-year period, and refer to the MR imaging appearances of the spine in a child with IJO who had previously received oral bisphosphonates.

## 2. Case Report

**Figure 1:**
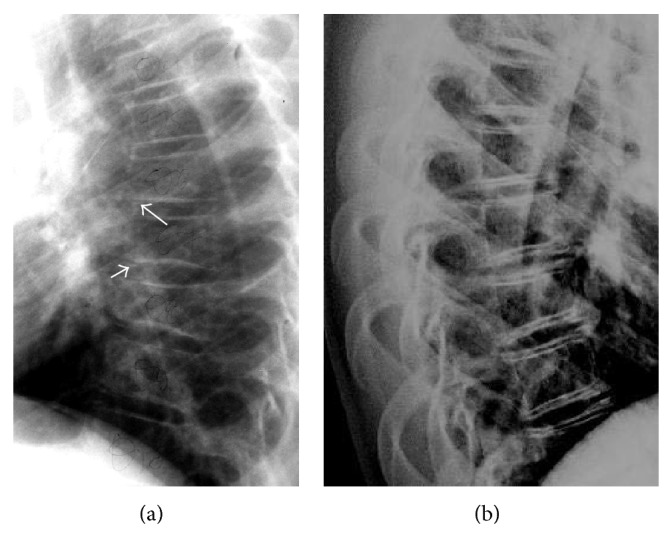
Lateral radiographs of the thoracic spine: (a) three months, (b) two years after bisphosphonate treatment ceased. In (a) there was osteopenic appearance, decreased height, and mild end-plate sclerosis of the thoracic vertebral bodies, whereas faint ossifications of the anterior endplates were seen (arrows). In (b) a typical “bone in bone” appearance of thoracic vertebral bodies was apparent.

**Figure 2:**
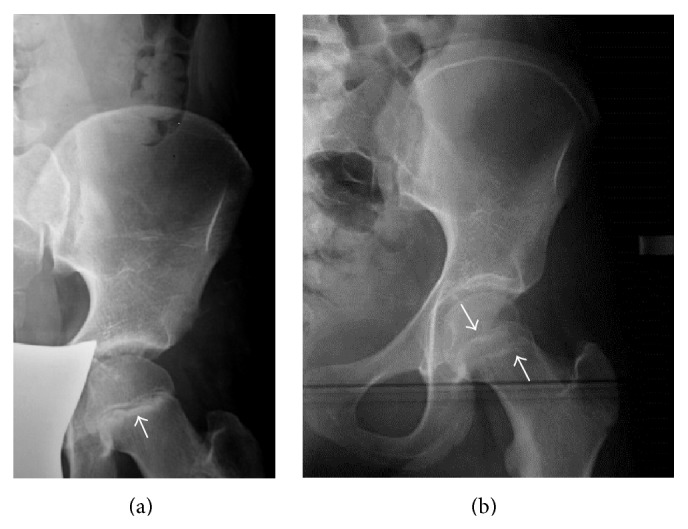
Frontal view of the left hip: (a) three months, (b) two years after bisphosphonate treatment ceased. In (a), dense metaphyseal lines (arrows) in the proximal femur and sclerosis of the acetabular rim were seen. In (b), there was mild sclerosis in both margins of closed physes (arrows). Three layers of bone were evident in iliac wing, with subchondral lucency of the outer layer and two layers in the ischial bone and the acetabulum.

A 10-year-old Caucasian female was referred to our pediatric clinic, first time in May 2009, for pain in the lower thoracic and upper lumbar spine. Both parents and two siblings were healthy. She was Turner stage II, with no difference between chronological and bone age. The diagnosis of IJO was made on the basis of (a) compression fracture of the tenth thoracic vertebral body, confirmed on radiographs, one year ago, without a history of trauma; (b) low bone mineral density, expressed by low *z*-score −2.3, as documented by dual-energy X-ray absorptiometry (DXA) of the L1–L4 lumbar spine; and (c) unremarkable results of an extensive investigation for low BMD, including screening for thyroid and rheumatologic diseases, osteogenesis imperfecta, hypercalcemia, vitamin D deficiency, and lysosomal storage diseases. Serum markers of bone turnover such as alkaline phosphatase, osteocalcin, and urinary hydroxyproline were within normal range. Because of continued skeletal pain and aiming to increase BMD and prevent further fractures the child received alendronate sodium orally, 70 mg per week for six months, in 2010, at the age of eleven, and her pain was improved. A lateral radiograph of the thoracic spine, three months after cessation of treatment, showed decreased height of lower thoracic vertebral bodies with barely discernible ossification of anterior epiphyseal plates for age (Figure 1(a)). A frontal radiograph of the pelvis showed symmetric sclerotic lines of the metaphyses of the proximal femurs and a rather sclerotic contour of both acetabula (Figure 2(a)). DXA, performed at the same time, revealed a *z*-score −1.8 determined at L1–L4 lumbar spine.

**Figure 3:**
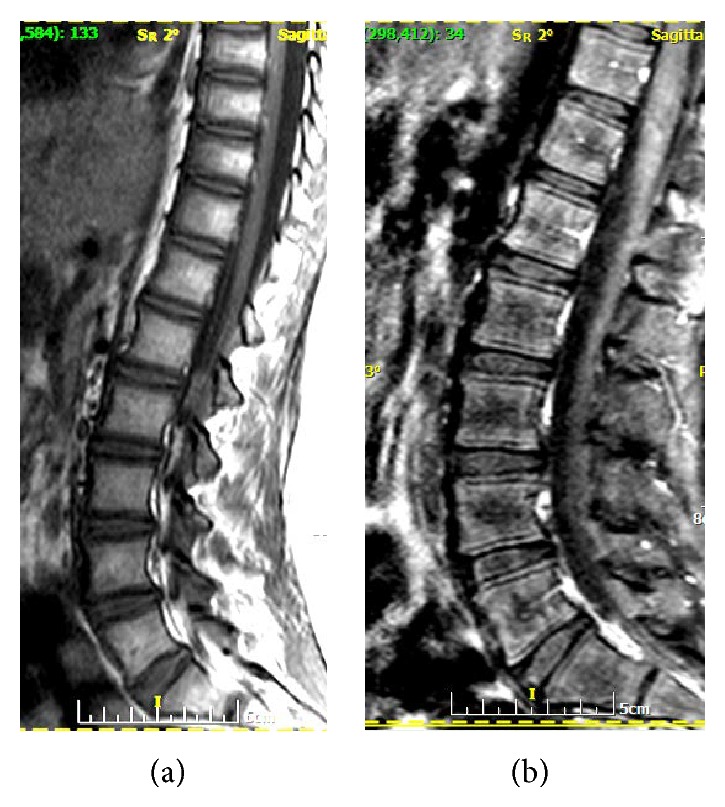
(a) A sagittal T1 SE image of the thoracolumbar spine showed mild hypointensity of the epiphyseal plates. (b) A sagittal T1 contrast-enhanced/fat-suppressed image demonstrated a double contour of vertebral bodies, with mild enhancement of the epiphyseal plates.

Two years after cessation of bisphosphonate treatment, in 2012, at the age of 13 and stage Turner IV, the patient suffered from relapsing back pain. Radiographs of the thoracic and lumbar spine exhibited a typical “bone in bone” appearance (Figure 1(b)). Repeat radiographs of the pelvis (Figure 2(b)) demonstrated three discrete parallel layers at iliac wings and a double contour of the acetabula and ischial bones. The growth plates were closed, and mild sclerotic margins of the closed proximal femoral physes were seen. Tubulation of the proximal femoral bones was normal. An MR imaging of the thoracolumbar spine was recommended to exclude other causes of back pain. Sagittal T2 and STIR images and axial T2 images were unremarkable, whereas sagittal T1 images disclosed a mild hypointensity of the epiphyseal plates (Figure 3(a)). It was noteworthy that T1-fat-suppressed/contrast-enhanced images disclosed a double contour of vertebral bodies corresponding to the “bone in bone” appearance on X-ray (Figure 3(b)). Repeat DXA revealed a *z*-score −1.1, for lumbar spine. In the subsequent months the patient noted decrease of pain although no medication was administered. In the subsequent two years, in 2014, radiological findings of spine and pelvis remained unchanged.

## 3. Discussion

Bisphosphonates are a group of agents which can bind to hydroxyapatite crystals on bone surfaces and inhibit bone resorption, by restraining osteoclast activity [1]. Numerous studies have documented the efficacy of bisphosphonates in adults, most commonly for the treatment of involutional osteoporosis, Paget's disease, hypercalcemia, and bone pain in malignant bone diseases. In pediatric populations, bisphosphonate administration, intravenous or oral, has been advocated in a growing number of disorders, mainly associated with generalized osteoporosis primary or secondary, but also in localized bone disease, soft tissue calcifications, and pathologic conditions with hypercalcemia, juvenile Paget's disease, Gaucher's disease, and polyostotic fibrous dysplasia [1, 2]. The use of bisphosphonates is well tolerated and results in decreased rate of bone fractures, relief from pain, and an increase of bone strength and *z*-score [1–3], without impact on bone growth [5, 6]. Most studies refer to their beneficial effect in patients with osteogenesis imperfecta, where they have reduced fracture rate by 50%, improved vertebral shape and BMD, and generally ameliorated the quality of life [1, 6]. Although no control studies are available in IJO, scarce case reports show that parenteral administration of bisphosphonates in patients with rapidly progressive IJO is well tolerated, reverts demineralization, and prevents skeletal deformities [2–5, 7]. Sebestyen et al. refer that children with IJO and vertebral collapse fractures, ceased pain medications and spine brace after intravenous administration of bisphosphonates [1]. In contrast to previous reports, our patient received oral bisphosphonates and she did for a shorter period of time. Sclerotic metaphyseal lines in long bones of rapid growth have been reported in children after bisphosphonate treatment [4–12]. These lines are thick and band-like in continuous use or thinner parallel lines, like “zebra lines” in intermittent use [7–9], and resemble the growth arrest lines, with each line corresponding to a cycle of treatment [10, 11]. They may persist for up to five years in the growing skeleton, due to increased half-life of bisphosphonates when incorporated into bones [8, 12], but fade over time. In our patient, there was one metaphyseal line initially, whereas in the subsequent two years two less dense metaphyseal lines were seen at the borders of the closed physes. Undertubulation of long bones has also been described after long-term, high-dose continuous bisphosphonate use [6] but was not expected in our patient who was treated only for a very short period. A “bone in bone” appearance of vertebrae, innominate bones, and carpal bones is equivalent to “zebra lines” [2, 4, 5, 7, 9]. van Meerten et al. noted a “picture frame” appearance of the vertebral bodies after continuous bisphosphonate administration and a “bone in bone” pattern after discontinuous use [7]. In our patient, the growing epiphyseal plates were barely visible three months after treatment ceased, whereas a more typical “bone in bone” pattern was revealed two years later which remained unchanged in two more subsequent years. Most patients with radiologic changes after bisphosphonate use reported in literature suffered from osteogenesis imperfecta or secondary osteoporosis and received parenteral or oral bisphosphonates in higher dosage and for a longer period than our patient [5–8]. Particularly all reported patients with IJO who presented thick metaphyseal bands and “bone in bone” vertebral bodies were treated with high-dose, intravenously administered bisphosphonates, unlike our patient with IJO who received alendronate orally. Therefore, it seems that radiologic changes after bisphosphonate treatment may occur irrespective of the pharmacological agent, the underlying cause, the route of administration, and the duration of treatment.

We should draw attention to the fact that, in the following months after treatment, the growing and faintly ossified epiphyseal plates might be misinterpreted as calcifications in the intervertebral disk. We observed that, within a two-year period, the initial sclerotic metaphysical lines of the proximal femurs decreased in density, in contrast to the more accentuated double contour of vertebral bodies and the appearance of discrete parallel lines at iliac crests and ischial bones; this evolution of radiographic changes in various bones of spine and pelvis, in the same patient, has not been adequately emphasized before. Sclerotic borders of the lumbar vertebrae should be also taken into account in the interpretation of *z*-score, since improved BMD may not actually reflect increased bone strength. It has been speculated that these bands are comprised of calcified cartilage, which undergoes progressive modeling over time [5, 6]. Osteomalacia does not appear to be a common finding with typical use. On the other hand, in patients with long-term, high-dose intravenous treatment sclerotic areas may be more brittle [8]. A “double contour” pattern of vertebrae seen only on contrast-enhanced/fat-suppressed T1-weighted MR images has not been previously described, to the best of our knowledge. Increased susceptibility of the sclerotic inner contour of the vertebral bodies when fat suppression is applied on a T1-weighted sequence, in association with mildly increased enhancement of the epiphyseal plates, may account for this “double contour” pattern. Mild hypointensity of the epiphyseal plates on unenhanced sagittal T1 images was attributed to more immature bone for age. We prefer to refer to a “double contour” to encompass both radiographic and MR imaging appearances.

The differential diagnosis of the radiographic “bone in bone pattern” in children includes osteopetrosis, treated rickets, heavy metal poisoning, oxalosis, hyperphosphatemia, and thorotrast intoxication [11, 13]. A double contour of the vertebral bodies on radiographs has also been observed in neonates either as a normal variant or after recovery of bone growth from a serious illness as equivalent to growth arrest lines [13].

*In conclusion*, since the use of bisphosphonates expands in a wide spectrum of pediatric disorders, physicians should familiarize with the expected imaging changes of bones.
